# Age and gender trends in insecticide-treated net use in sub-Saharan Africa: a multi-country analysis

**DOI:** 10.1186/s12936-018-2575-z

**Published:** 2018-11-14

**Authors:** Bolanle Olapeju, Ifta Choiriyyah, Matthew Lynch, Angela Acosta, Sean Blaufuss, Eric Filemyr, Hunter Harig, April Monroe, Richmond Ato Selby, Albert Kilian, Hannah Koenker

**Affiliations:** 1 PMI VectorWorks Project, Johns Hopkins Center for Communication Programs, School of Public Health, 111 Marketplace, Baltimore, MD 21202 USA; 2 Department of Population, Family and Reproductive Health, Johns Hopkins University Bloomberg School of Public Health, Baltimore, USA; 3 PMI VectorWorks Project, Tropical Health LLP, Montagut, Spain

**Keywords:** Insecticide-treated nets, Use, Household supply, Age, Gender, Household members, Sub-Saharan Africa

## Abstract

**Background:**

The degree to which insecticide-treated net (ITN) supply accounts for age and gender disparities in ITN use among household members is unknown. This study explores the role of household ITN supply in the variation in ITN use among household members in sub-Saharan Africa.

**Methods:**

Data was from Malaria Indicator Surveys or Demographic and Health Surveys collected between 2011 and 2016 from 29 countries in sub-Saharan Africa. The main outcome was ITN use the previous night. Other key variables included ITN supply (nets/household members), age and gender of household members. Analytical methods included logistic regressions and meta-regression.

**Results:**

Across countries, the median (range) of the percentage of households with enough ITNs was 30.7% (8.5–62.0%). Crude analysis showed a sinusoidal pattern in ITN use across age groups of household members, peaking at 0–4 years and again around 30–40 years and dipping among people between 5–14 and 50+ years. This sinusoidal pattern was more pronounced in households with not enough ITNs compared to those with enough ITNs. ITN use tended to be higher in females than males in households with not enough ITNs while use was comparable among females and males in households with enough ITNs. After adjusting for wealth quintile, residence and region, among households with not enough ITNs in all countries, the odds of ITN use were consistently higher among children under 5 years and non-pregnant women 15–49 years. Meta-regressions showed that across all countries, the mean adjusted odds ratio (aOR) of ITN use among children under 5 years, pregnant and non-pregnant women aged 15–49 years and people 50 years and above was significantly higher than among men aged 15–49 years. Among these household members, the relationship was attenuated when there were enough ITNs in the household (dropping 0.26–0.59 points) after adjusting for geographical zone, household ITN supply, population ITN access, and ITN use:access ratio. There was no significant difference in mean aOR of ITN use among school-aged children compared to men aged 15–49 years, regardless of household ITN supply.

**Conclusions:**

This study demonstrated that having enough ITNs in the household increases level of use and decreases existing disparities between age and gender groups. ITN distribution via mass campaigns and continuous distribution channels should be enhanced as needed to ensure that households have enough ITNs for all members, including men and school-aged children.

## Background

According to the World Malaria Report, there were an estimated 216 million cases of malaria globally in 2016 while the estimated number of malaria deaths was 445,000 in 2016 [1]. Africa continues to carry a disproportionately higher share of the global malaria burden as 90% of malaria cases and deaths occur in this continent with 15 countries in sub-Saharan Africa accounting for 80% of the global malaria burden [1]. The World Health Organization (WHO) recommends the use of insecticide-treated nets (ITNs) as a key element of vector control by all individuals at risk of malaria, and distribution of free ITNs is a core intervention in national malaria control strategies of all sub-Saharan Africa countries [2]. In an effort to achieve universal coverage, i.e., universal access to and use of ITNs by populations at risk of malaria [3], over 800 million nets have been delivered in sub-Saharan Africa between 2011 and 2016, mostly under universal coverage campaigns [1]. This investment has resulted in an increased proportion of Africans in malaria-endemic areas who slept under an ITN, from 2010 30%, to 2016 54% [1]. The concept of universal access and indicators used to measure it are based on the assumption that each ITN protects two people [1]. To further improve ITN coverage in Africa, gaps in ITN access as well as ITN use need to be explored and addressed [4].

Recent studies have shown that the major driver of ITN use is access, as one cannot use an ITN unless there is one available for use [5–8]. After ITN access has been addressed, individual level factors, including age and gender of household members, have also been associated with ITN use. Studies across Africa demonstrate that ITN use is typically higher among females compared to males [9]. ITN use is also correlated with age [10] and has been shown to be higher in certain age groups, e.g., infants [11] or children under 5 years of age [12] compared to older children aged 5–14 years and adolescents and young adults aged 15–24 [13, 14]. The association of age with ITN use also seems to be moderated by gender, such that men, older children and teenagers were less likely to sleep under an ITN compared to women and children under 5 years old [15]. It is unclear whether certain household members are prioritized only because the number of nets in the household is not enough. Thus, the supply of nets in the household might be the reason for the age/gender disparities in ITN use.

This paper explores to what extent ITN supply (having enough nets for household members) accounts for age and gender disparities in IT N use among household members in sub-Saharan Africa. ITN use has been shown to increase dramatically in all age groups and gender following mass free distribution of ITN [13, 16] suggesting that certain household members are prioritized for ITN use when there are not enough ITNs in the household. The relationships between ITN supply, household members and ITN use are worth exploring to understand whether improving supply of ITNs in a household might reduce age and gender disparities in ITN use.

## Methods

This study analyses secondary data from recent national surveys in sub-Saharan Africa.

Data from recent (conducted between 2011 and 2016) Malaria Indicator Surveys (MIS) or Demographic and Health Surveys (DHS) among countries in sub-Saharan Africa, were included in the analysis. Recent surveys were defined as those conducted between 2011 and 2016. The most recent publicly available MIS or DHS data from a total of 29 malaria endemic countries (Namibia was excluded given its limited malaria risk [1]) were downloaded with permission from the DHS Programme website, http://www.dhsprogram.com.

The countries were categorized into 3 geographical zones, Central, East and West Africa, based on the United Nations geoscheme for Africa [17]. East Africa region included 10 countries (34.5%), Central Africa, 7 countries (24.1%) and West Africa, 12 countries (41.4%).

The main outcome of the study is use of an ITN the previous night and this was calculated for each de facto member of the household, i.e., all those present in the house the previous night, as recommended by WHO’s Roll Back Malaria Monitoring and Evaluation Reference Group (MERG) [7, 18]. A main predictor variable was household ITN supply and this was defined as the number of ITNs present in the household divided by the *de jure* household members and was further dichotomized into ‘not enough’ (ITN: person ratio of less than 0.5) *versus* ‘enough’ (ITN: person ratio of 0.5 or more equivalent to one ITN for every 2 people). The other main predictor variables of interest included gender (male *versus* female) and age (categorized in 5–10 year increments (0–4, 5–9, 10–14, 15–19, 20–29, 30–39, 40–49, 50–59, and 60+ years) of de facto household members. In addition, a composite variable called ‘demographic group’ variable was created based on age, gender and pregnancy status of the de facto household members. The following demographic groups were defined: children under 5 years old, school-aged children 5–14 years, women aged 15–49 years who were currently pregnant, women aged 15–49 years who were not currently pregnant, men aged 15–49 years (reference group) and adults aged 50 years or more.

Other socio-demographic variables included household wealth quintile based on the standard DHS wealth index determined by principal component analysis on household assets, residence (urban/rural), and region (sub-national administrative divisions for each country). Two contextual variables included in the analysis include population level ITN access, and use given access (use:access ratio). The population ITN access indicator for each country was calculated according to MERG guidance by dividing the potential ITN users (number of ITNs in the household multiplied by 2) by the number of de facto members for each household, setting the result to 1 if there were more potential users than de facto members, and determining the overall sample mean of that fraction [7]. To assess whether people who have ITNs actually use them, the ratio of population ITN use to population ITN access was calculated.

All analysis was limited to households with at least one ITN. First, plots of ITN use by age and gender of de facto household members, stratified by household ITN supply were constructed for each country separately. Then, multivariable logistic regressions were conducted for each country, stratified by household ITN supply, to explore differences in ITN use among demographic groups, controlling for household wealth quintile, residence and region. Next, to synthesize the findings across all countries, a meta-regression was conducted to explore the mean adjusted odds ratio (aOR) of ITN use across demographic groups across all 29 countries. Each country was stratified by household ITN supply for a total sample size of 58. Plots of the mean aOR and 95% confidence interval (CI) of ITN use among demographic groups stratified by ITN supply were constructed over all countries and also by the 3 geographic zones (Central, East and West Africa). The model included the following country-level covariates: geographical zone, household ITN supply, population ITN access and ITN use:access ratio. To account for different sample size of each country, the number of de facto populations in households with at least one ITN was used as a probability weight.

Data management and analysis was done using Stata version 14 (Stata Corporation, College Station, TX, USA) and Excel 2016 (Microsoft Corp, Seattle, WA, USA). All country-level analyses used sample weights to adjust for DHS sample design and individual response rate [19].

## Results

Table 1 presents the proportion of households with enough ITNs and population-level ITN access and use:access ratio for each survey. Across countries, the median (range) of the percentage of households with enough ITNs was 30.7% (8.5–62.0%). The median (range) of the percentage of households with enough ITNs was 14.5% (8.5–24.3%) in Central; 38.4% (22.7–62.0%) in East Africa; and, 30.7% (9.3–56.7%) in West Africa. In only 3 countries did more than 50% of households own enough ITNs: Uganda (62.0%), Senegal (56.7%) and Ghana (50.3%). Similarly, the median (range) of the percentage of the de facto population with access to an ITN in their household was 26.9% (19.7–61.2%) in Central; 55.9% (37.2–78.8%) in East; and, 49.0% (25.3–75.7%) in West Africa. Overall, the proportion of the population that used an ITN the previous night was greater than 50% in only 8 countries (Madagascar, Rwanda, Uganda, Democratic Republic of Congo, Benin, Burkina Faso, Mali, Senegal). ITN use:access ratio varied widely across the countries from 0.23 in Zimbabwe to 1.15 in Congo-Brazzaville.

**Table 1:** List of countries and key insecticide-treated net indicators

Country	Survey	Year	% of households with enough ITNs^a^	% of *de facto* population with ITN access	% of *de facto* population that used an ITN the previous night	Use:access ratio
Central Africa						
Angola	DHS	2015–16	10.9	19.7	17.6	0.89
Burundi	MIS	2012	23.9	46.0	48.6	1.06
Cameroon	DHS	2011	8.5	20.9	14.8	0.71
Chad	DHS	2014–15	40.8	61.2	33.3	0.54
Congo Brazzaville	DHS	2011–12	10.4	22.6	26.0	1.15
Democratic Republic of Congo	DHS	2013–14	24.3	46.5	50.2	1.08
Gabon	DHS	2012	14.5	26.9	26.7	0.99
East Africa						
Kenya	MIS	2015	40.1	52.5	47.6	0.91
Madagascar	MIS	2016	43.1	62.1	68.2	1.10
Malawi	DHS	2015–16	22.7	38.8	33.9	0.87
Mozambique	DHS	2015	38.4	53.8	45.4	0.84
Rwanda	DHS	2014–15	42.2	63.8	61.4	0.96
Tanzania	DHS	2015–16	37.2	55.9	49.0	0.88
Uganda	MIS	2014–15	62.0	78.8	68.6	0.87
Zambia	DHS	2013–14	25.0	65.0	56.9	0.88
Zimbabwe	DHS	2015	26.1	37.2	8.5	0.23
West Africa						
Benin	DHS	2011–12	43.3	64.0	62.6	0.98
Burkina Faso	MIS	2014	47.4	71.2	67.0	0.94
Cote D’Ivoire	DHS	2011	30.7	49.0	33.2	0.68
Gambia	DHS	2013	20.1	45.3	36.9	0.82
Ghana	MIS	2016	50.3	65.8	41.7	0.63
Guinea	DHS	2012	9.3	25.3	18.9	0.75
Liberia	MIS	2016	23.5	41.5	39.2	0.94
Mali	MIS	2015	37.6	69.5	63.8	0.92
Niger	DHS	2012	14.4	37.3	13.8	0.37
Nigeria	MIS	2015	34.4	54.7	37.3	0.68
Senegal	cDHS	2016	56.7	75.7	63.1	0.83
Sierra Leone	MIS	2016	14.6	37.1	38.6	1.04
Togo	DHS	2013–14	32.5	48.8	33.6	0.69

*DHS* Demographic Health Survey, *ITN* insecticide-treated nets, *MIS* Malaria Indicator Survey

^a^A household supply of at least 0.5 net per person

**Fig. 1:**
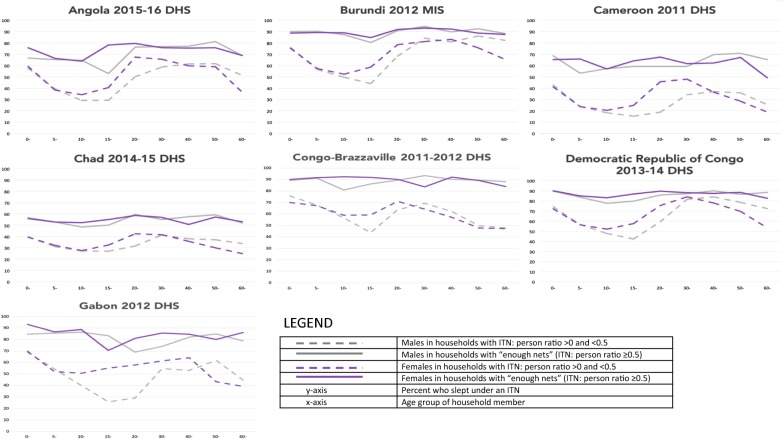
Insecticide-treated net use by insecticide-treated net supply, age and gender in Central Africa

**Fig. 2:**
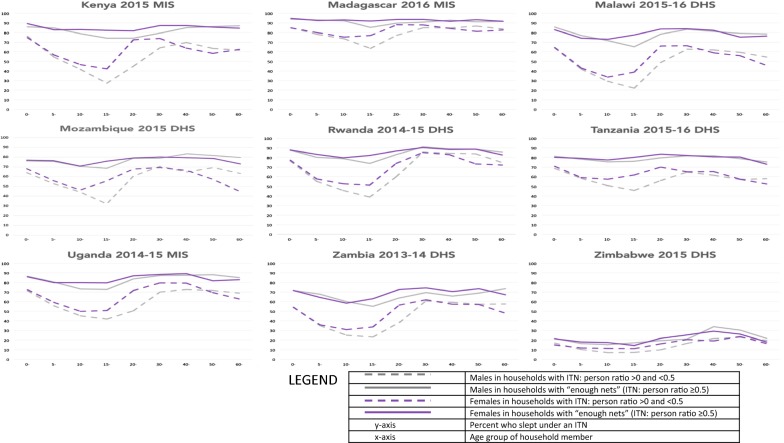
Insecticide-treated net use by insecticide-treated net supply, age and gender in East Africa

**Fig. 3:**
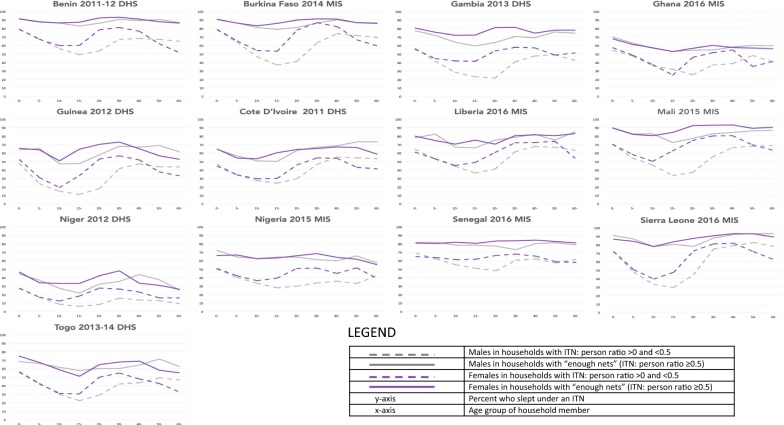
Insecticide-treated net use by insecticide-treated net supply, age and gender in West Africa

Figures 1, 2, 3 highlight country-level population ITN use stratified by ITN supply, age and gender in Central (Fig. 1), East (Fig. 2) and West (Fig. 3) Africa. In all countries, regardless of age and gender, ITN use was higher among people in households with enough ITNs compared to those in households with not enough ITNs. For people from households with not enough ITNs, ITN use showed a sinusoidal pattern, peaking at 0–4 years and again around 30–40 years and dipping among people between 5–14 and 50+ years. This sinusoidal pattern was less pronounced in households with enough ITNs. In households with not enough ITNs, ITN use was higher in females compared to males in many age groups. Among people living in households with enough ITNs, use was more comparable among males and females in all age groups.

Table 2 presents the aOR of ITN use the previous night among demographic groups (reference group: men 15–49 years) stratified by household ITN supply and controlling for household wealth index, household residence and region.

**Table 2:** Logistic regression of insecticide-treated net use among demographic groups (reference: men aged 15–49 years) stratified by insecticide-treated net supply, adjusted for wealth index, residence (urban/rural), and region

Country	aOR^a^of ITN use among household members by household ITN supply									
	Households with not enough ITNs (ref: male 15–49 years)					Households with enough ITNs (ref: male 15–49 years)				
	Children under 5 years	School-aged (5**–**14 years)	Female 15**–**49 years		50 + years	Children under 5 years	School-aged (5**–**14 years)	Female 15**–**49 years		50+ years
			Not pregnant	Currently pregnant				Not pregnant	Currently pregnant	
East Africa										
Madagascar	1.63*	0.93	1.76*	1.23	1.53*	1.82*	1.46*	1.41*	1.99*	1.21
Mozambique	1.48*	0.71*	1.48*	1.76*	1.12	0.99	0.80*	1.12	2.42*	1.12
Zimbabwe	1.22*	0.71*	1.33*	1.07	1.65*	0.97	0.73*	1.10	0.46*	1.08
Zambia	1.42*	0.56*	1.41*	1.48*	1.51*	1.37*	0.89	1.31*	2.03*	1.33*
Malawi	2.01*	0.66*	1.65*	1.51*	1.31*	1.73*	0.88*	1.43*	1.05	1.07
Rwanda	1.68*	0.58*	1.43*	3.55*	1.69*	1.48*	0.85*	1.29*	2.31*	1.38*
Tanzania	1.83*	1.02	1.60*	1.66*	1.08	1.21*	0.98	1.20*	1.08	0.98
Uganda	1.98*	0.85	1.80*	2.37*	1.70*	1.27*	0.76*	1.28*	1.61*	1.10
Kenya	3.2*	1.01	1.9*	3.57*	1.64*	2.04*	1.28*	1.59*	1.54	1.71*
Central Africa										
Angola	1.45*	0.57*	1.61*	2.26*	1.24	1.13	0.78*	1.41*	2.56*	1.23
Burundi	1.43*	0.55*	1.30*	2.64*	1.72*	1.08	1.07	1.13	2.74	1.14
Cameroon	2.34*	0.89	1.94*	2.89*	1.10	1.52*	0.98	1.21	0.76	0.98
Chad	1.56*	0.94	1.47*		1.08	1.22*	0.92	1.14*		1.14
Congo-Brazzaville	1.70*	1.1	1.22*	1.36	0.62*	0.93	0.90	0.90	1.50	0.77
DRC	1.45*	0.60*	1.57*	1.78*	1.26*	1.5*	0.79*	1.28*	1.70	1.13
Gabon	3.4*	1.49*	2.24*	2.28*	1.38*	2.8*	2.40*	1.45*	1.75	1.48
West Africa										
Benin	2.52*	1.20*	2.11*	4.27*	1.13*	1.54*	1.04	1.57*	2.00*	1.06
Burkina Faso	3.2*	1.22*	2.94*	4.24*	1.87*	1.82*	1.05	1.72*	1.97*	1.26*
Gambia	3.18*	1.58*	2.65*	3.21*	2.18*	2.25*	1.37*	1.89*	4.36*	1.94*
Ghana	2.60*	1.46*	1.80*	2.16*	1.35	1.82*	1.16	1.17	1.79*	1.01
Guinea	2.74*	0.77*	2.72*	3.45*	1.92*	1.49*	1.12	1.80*	1.37	1.38*
Cote D’Ivoire	1.27*	0.69*	1.47*	1.18	1.46*	0.94	0.60*	1.06	1.51*	1.00
Liberia	1.60*	0.94	1.72*	1.86*	1.72*	1.05	0.84	1.07	1.23	1.17
Mali	2.65*	1.24*	3.36*	3.66*	2.37*	2.20*	1.15	2.59*	2.65*	1.87*
Niger	3.81*	1.57*	3.18*	3.00*	1.43*	2.03*	1.09	1.52*	1.59	0.94
Nigeria	2.20*	1.30*	2.04*	2.72*	1.54*	1.28*	1.02	1.19*	1.25	0.99
Senegal	1.66*	1.20*	1.66*		1.19	1.47*	1.33*	1.52*		1.30*
Sierra Leone	1.86*	0.56*	1.90*	2.05*	2.03*	1.08	0.71*	1.37*	2.03	1.80*
Togo	2.56*	1.13*	1.84*	1.93*	1.39*	1.63*	1.13	1.17*	1.37	0.99

Data not available

*aOR* adjusted odds ratio, *ITN* insecticide-treated net

^a^Adjusted for wealth index, residence (urban/rural), and region

* Significant at *p* value < 0.05

Among households with not enough ITNs, two demographic groups: children under 5 years and non-pregnant women had consistent significantly higher odds of ITN use compared to men aged 15–49 years in all countries. The median (range) aOR of ITN use among children under 5 years old in all 29 countries was 1.86 (1.22–3.81). Non-pregnant women in all 29 countries had a median (range) aOR of 1.76 (1.22–3.36). In addition, pregnant women in all 27 countries with available data had a median (range) aOR of 2.26 (1.48–4.27), although the aOR was not statistically significant in Zimbabwe, Ivory Coast, Madagascar, and Congo-Brazzaville. Children aged 5–14 years had a median (range) aOR of 0.94 (0.55–1.58); the aOR was significantly lower in 11 countries, significantly higher in 10 countries and not statistically significant in 8 out of 29 countries.

Among households with enough ITNs, the disparities in ITN use across demographic groups was attenuated. There was no demographic group with significantly higher odds of ITN use across all countries. The median (range) aOR of ITN use among children under 5 years old was 1.48 (0.93–2.80) although the aOR was not statistically significant in 8 and significantly higher in 21 of the 29 countries. Pregnant women had a median (range) aOR of ITN use of 1.29 (0.90–2.59). Similarly, the aOR was not statistically significant in eight countries and significantly higher in 21 countries of the 29 countries. Among pregnant women, the median (range) aOR of ITN use was 1.75 (0.46–4.36) although the aOR was significantly lower in Zimbabwe, not statistically significant in 14 countries and significantly higher in 12 of the 27 countries with available data. Children aged 5–14 years had a median (range) aOR of 0.98 (0.60–2.40), the aOR was significantly lower in 9 countries, significantly higher in 5 countries and not statistically significant in 15 countries.

**Fig. 4:**
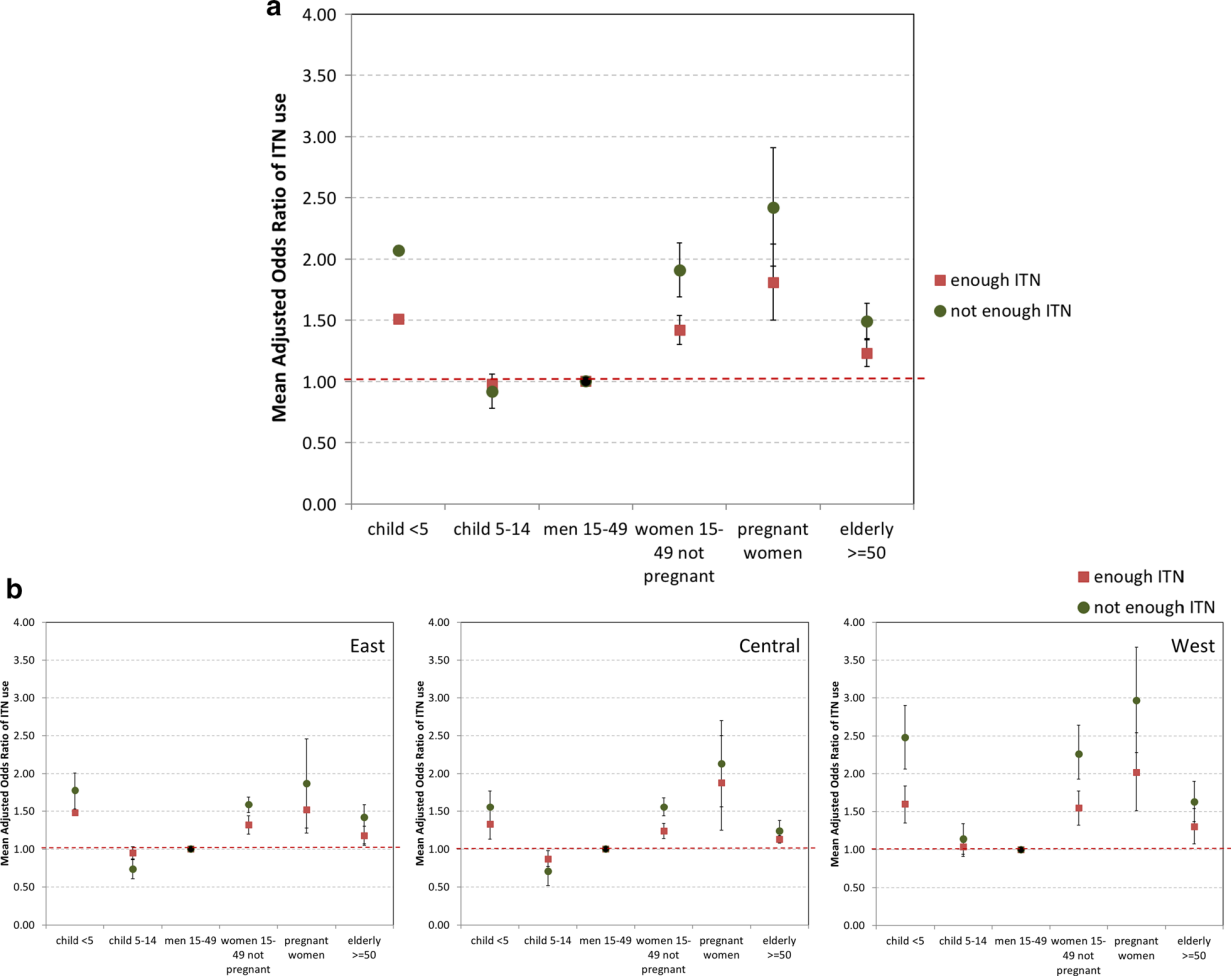
Mean adjusted odds ratios for insecticide-treated net use among demographic groups (reference group: men aged 15–49), by insecticide-treated net supply, overall (**a**) and by geographic region (**b**)

Figure 4 presents results of the meta-regression of the aORs of ITN use among demographic groups, stratified by ITN supply across all 29 countries, and in addition, for each geographic zone. Overall, the mean aOR of ITN use was significantly higher among children under 5 years, pregnant and non-pregnant women aged 15–49 years and people 50 years and above compared to the reference group of men aged 15–49 years. Also, the differences in ITN use across demographic groups tended to be reduced when there were enough ITNs. In addition, for children under 5 years, pregnant and non-pregnant women aged 15–49 years and people 50 years and above, the aORs of ITN use were higher in households with enough ITNs compared to households with not enough ITNs. There was no significant difference in mean aOR of ITN use among school-aged children compared to men aged 15–49 years, regardless of household ITN supply. This trend was seen over all countries and across the 3 geographic zones. Of note, the variation in mean aOR of ITN use across household members was most pronounced in West compared to East or Central Africa.

The meta-regression results in Table 3 highlight the influence of country-level ITN supply, population ITN access, ITN use:access ratio and geographic region on the mean aOR of ITN use for demographic groups across all 29 countries. The effect sizes shown in the Table represent the change in mean aOR per unit change of each covariate, holding others constant. Thus, the mean aOR is treated as a continuous variable in this analysis. For example, the mean aOR of ITN use among children under 5 years reduces by 0.59 points in households with not enough compared to enough ITN supply while each per cent increase in population ITN access has minimal effect on the mean aOR of ITN use. In general, the results confirm earlier findings, as the mean aORs of ITN use decreased (dropping by 0.26–0.59 points) among almost all demographic groups compared to men age 15–49 years when there are enough ITNs in the household compared to households with not enough ITNs. The only exception was the group children 5–14 years for whom the mean aOR did not change with household ITN supply. The level of population access to ITNs at the time of the survey (as shown in Table 1) did not have any impact on the mean aOR of ITN use among household members, again with the exception of children 5–14 years for whom the mean aOR increased by 0.06 for each 10% increase in population access. Changes in use-to-access ratio did not significantly contribute to differentials in the mean aOR of ITN use across demographic groups. As was suggested in Fig. 4, the mean aOR of ITN use for household members, except the 50 years and over, was significantly higher in West compared to the East Africa.

**Table 3:** Adjusted linear regression coefficients for mean adjusted odds ratios of insecticide-treated net use

Independent variable	Adjusted linear regression coefficients by demographic group^a^				
	Children under 5 years	School-aged (5–14 years)	Female 15–49 years		50+ years
			Not pregnant	Pregnant	
Household ITN supply enough vs not enough	− 0.568*	0.524	− 0.497*	− 0.591*	− 0.258*
Population access in %^b^	− 0.0001	0.006*	− 0.000	0.013	− 0.005
Use:access ratio^b^	− 0.195	− 0.399	0.221	1.072	0.680
Central Africa vs East	− 0.168	0.036	− 0.040	0.389	− 0.195
West Africa vs East	0.424*	0.231*	0.479*	0.779*	0.179
R squared	0.384	0.332	0.463	0.337	0.328

*ITN* insecticide-treated net

^a^Covariates included in the model: household ITN supply, population ITN access and geographic zone

^b^Variable shown in Table 1

* Significant at p-value < 0.05

## Discussion

This study demonstrated that regardless of setting and across a large number of countries, the groups most vulnerable to malaria are preferentially being covered, per WHO recommendations that pregnant women and infants in malaria-endemic areas use ITNs. It also suggests that ITNs are not hoarded by heads of households but used among household members, depending on household supply. The study showed that having enough ITNs in the household increases level of use and decreases existing disparities between age and gender groups. ITN use was consistently higher among people in households with enough compared to not enough nets. The role of ITN supply on use is important given the WHO target of 85% coverage of key malaria interventions, including ITN use by all people at risk of malaria, and the WHO recommendation of one ITN for every two people at risk of malaria [1]. Many countries struggle to meet this target among all households but have been able to achieve the target among households with enough ITNs. This suggests that people are typically willing to use ITNs but need to have enough ITNs to increase and sustain ITN use. Thus, increasing the household supply of ITNs improves use among members. These findings provide further evidence that the main barrier to ITN use is perhaps insufficient access and to a lesser degree unwillingness to use ITNs [5, 7, 8].

Our findings highlight existing disparities in ITN use among household members, corroborating previous research [10–13, 15]. In most of sub-Saharan Africa, households rightfully prioritize children under 5 years as well as pregnant women, especially when there is not enough ITN supply. Children under 5 years and pregnant women of reproductive age may be more likely to sleep under an ITN because, in many settings, those children share sleeping spaces with their mothers or adolescent female siblings [20]. It may also be due to the ITN interventions of the last few decades targeting pregnant women and children under 5 years old [9]. While pregnant women and young children are biologically vulnerable to malaria, there are negative side effects with only prioritizing them for ITN use. Contraction of malaria by other household members still has unwelcome health, social and financial consequences for the family, hence the emphasis on universal coverage [9].

The role of ITN supply on disparities in ITN use among household members is a novel addition of this study to the existing literature. Pregnant women, children under 5 years old, women aged 15–49 years, and those over 50 years were still more likely to have used an ITN the previous night than men but having enough ITNs within the household reduced the gaps in ITN use across these groups. However, school-children aged 5–14 years were among the least prioritized in households, regardless of household ITN supply. Studies have found that school-aged children had the highest prevalence of malaria infection but were most likely to have asymptomatic infection, thus serving as an under recognized reservoir of malaria infection [21, 22]. Protecting this age group with ITNs would reduce adverse health outcomes, such as anaemia and mortality, and educational outcomes such as school absenteeism and lower cognitive function [23]. In addition, protecting this age group with ITNs could protect the rest of the population from malaria transmission. As recommendations shift from covering vulnerable populations to universal coverage, there is a need to ensure that households have enough nets to eliminate disparities in ITN use among members. Mass distribution campaigns have been a major source of ITN supply in households, however, gaps in ITN coverage have been demonstrated between mass campaign cycles. Continuous distribution of ITNs through antenatal care, immunization services, communities, and schools has been recommended by WHO to complement mass campaigns and ensure universal coverage of ITNs, particularly antenatal care clinic and expanded programmes on vaccination distribution [3]. Continuous community-based [24, 25] and school-based ITN distribution [24, 26] has been shown to improve ITN ownership and access. However, although continuous antenatal care (ANC) and expanded programme on immunization (EPI) distribution systems targeting biologically vulnerable groups, such as children under 5 years and pregnant women, are supposed to be in place in almost every country, these are often low functioning, contributing to gaps in net access [25]. Efforts to improve the quality of existing distribution channels may involve ensuring complete household registration, enhancing data and communication campaigns to promote acceptability and uptake of distribution channels.

There are some limitations within this study. The analysis assumes that all ITNs included in the indicator of ITN supply in the household are all hung or usable. The study also uses slightly different denominators for the ITN indicators. Specifically, ITN supply is calculated from the *de jure* household members while ITN use is calculated from de facto members. This may be important in instances where the de facto and *de jure* members are markedly different. Seasonality of ITN use [27] is one of most important factors of ITN use but was not accounted for in this analysis. Research has shown seasonal variations in ITN use in sub-Saharan Africa, which may explain some of the differences in ITN use across countries as MIS and DHS surveys are usually conducted in different seasons. Typically, MIS is conducted during/at the end of rainy season while the DHS can be done any season. Given that ITN use is higher in the rainy season and immediately thereafter when malaria transmission is at a peak [28, 29], ITN use is higher in MIS survey countries than in DHS countries. Also, the timing of the most recent ITN mass campaigns was not accounted for in the analysis. Mass campaigns that are closely followed by household surveys generally show higher levels of population ITN access, which in turn makes high levels of ITN use feasible [13, 16]. In addition, the data analysed are cross-sectional in nature and thus do not permit causal inferences.

Finally, the study found some differences in ITN use among household members across the geographic zones explored. However due to the country eligibility criteria, not all countries within the three geographic regions are explored. Thus, regional differences in ITN use should be interpreted with caution. Also, malaria control research and programmatic efforts are also needed to understand the specific country level contextual factors that may explain trends in ITN access and use. For example, Zimbabwe has low levels of ITN use even among people in households with enough ITNs, and this may be related to national level indoor residual spraying interventions, resulting in a lower net use culture [30].

## Conclusion

This study explored the role of ITN supply on ITN use among household members. The findings suggest that having enough ITNs in the household increases level of use and decreases existing disparities between age and gender groups. School-aged children were also consistently the least prioritized, regardless of a household’s ITN supply. ITN distribution via mass campaigns, ANC and EPI, school and community channels should be enhanced as needed in order to ensure that households have enough ITNs for all members, including men and school-aged children.

## Abbreviations

aOR: adjusted odds ratio
ANC: antenatal care
CI: confidence interval
DHS: Demographic Health Survey
EPI: expanded programme on immunization
ITN: insecticide-treated nets
MERG: Monitoring and Evaluation Reference Group
MIS: Malaria Indicator Survey
WHO: World Health Organization

## References

[1] WHO (2017). World malaria report 2017.

[2] Sexton AR (2011). Best practices for an insecticide-treated bed net distribution programme in sub-Saharan eastern Africa. Malar J..

[3] WHO (2017). Achieving and maintaining universal coverage with long-lasting insecticidal nets for malaria control.

[4] van Eijk AM, Hill J, Alegana VA, Kirui V, Gething PW, ter Kuile FO (2011). Coverage of malaria protection in pregnant women in sub-Saharan Africa: a synthesis and analysis of national survey data. Lancet Infect Dis..

[5] Eisele TP, Keating J, Littrell M, Larsen D, Macintyre K (2009). Assessment of insecticide-treated bednet use among children and pregnant women across 15 countries using standardized national surveys. Am J Trop Med Hyg.

[6] Graves PM, Ngondi JM, Hwang J, Getachew A, Gebre T, Mosher AW (2011). Factors associated with mosquito net use by individuals in households owning nets in Ethiopia. Malar J..

[7] Koenker H, Kilian A (2014). Recalculating the net use gap: a multi-country comparison of ITN use versus ITN access. PLoS ONE.

[8] Bhatt S, Weiss DJ, Mappin B, Dalrymple U, Cameron E, Bisanzio D (2015). Coverage and system efficiencies of insecticide-treated nets in Africa from 2000 to 2017. eLife.

[9] Garley AE, Ivanovich E, Eckert E, Negroustoueva S, Ye Y (2013). Gender differences in the use of insecticide-treated nets after a universal free distribution campaign in Kano State, Nigeria: post-campaign survey results. Malar J..

[10] Ng’ang’a PN, Jayasinghe G, Kimani V, Shililu J, Kabutha C, Kabuage L (2009). Bed net use and associated factors in a rice farming community in Central Kenya. Malar J..

[11] Larson PS, Minakawa N, Dida GO, Njenga SM, Ionides EL, Wilson ML (2014). Insecticide-treated net use before and after mass distribution in a fishing community along Lake Victoria, Kenya: successes and unavoidable pitfalls. Malar J..

[12] Fokam EB, Kindzeka GF, Ngimuh L, Dzi KT, Wanji S (2017). Determination of the predictive factors of long-lasting insecticide-treated net ownership and utilisation in the Bamenda Health District of Cameroon. BMC Public Health..

[13] Loha E, Tefera K, Lindtjorn B (2013). Freely distributed bed-net use among Chano Mille residents, south Ethiopia: a longitudinal study. Malar J..

[14] Noor AM, Kirui VC, Brooker SJ, Snow RW (2009). The use of insecticide treated nets by age: implications for universal coverage in Africa. BMC Public Health..

[15] Babalola S, Ricotta E, Awantang G, Lewicky N, Koenker H, Toso M (2016). Correlates of intra-household ITN use in Liberia: a multilevel analysis of household survey data. PLoS ONE.

[16] Finlay AM, Butts J, Ranaivoharimina H, Cotte AH, Ramarosandratana B, Rabarijaona H (2017). Free mass distribution of long lasting insecticidal nets lead to high levels of LLIN access and use in Madagascar, 2010: a cross-sectional observational study. PLoS ONE.

[17] Statistics Division UN (1999). Standard country or area codes for statistics use.

[18] MEASURE Evaluation, MEASURE DHS, President’s Malaria Initiative, Roll Back Malaria Partnership, UNICEF, WHO (2013). Household survey indicators for malaria control.

[19] Rutstein SO, Rojas G (2006). Guide to DHS statistics.

[20] Toé LP, Skovmand O, Dabiré KR, Diabaté A, Diallo Y, Guiguemdé TR (2009). Decreased motivation in the use of insecticide-treated nets in a malaria endemic area in Burkina Faso. Malar J..

[21] Walldorf JA, Cohee LM, Coalson JE, Bauleni A, Nkanaunena K, Kapito-Tembo A (2015). School-age children are a reservoir of malaria infection in Malawi. PLoS ONE.

[22] Pullan RL, Bukirwa H, Staedke SG, Snow RW, Brooker S (2010). Plasmodium infection and its risk factors in eastern Uganda. Malar J..

[23] Nankabirwa J, Brooker SJ, Clarke SE, Fernando D, Gitonga CW, Schellenberg D (2014). Malaria in school-age children in Africa: an increasingly important challenge. Trop Med Int Health..

[24] Zeger de Beyl C, Kilian A, Brown A, Sy-Ar M, Selby RA, Randriamanantenasoa F (2017). Evaluation of community-based continuous distribution of long-lasting insecticide-treated nets in Toamasina II District, Madagascar. Malar J..

[25] Kilian A, Woods Schnurr L, Matova T, Selby RA, Lokko K, Blaufuss S (2017). Evaluation of a continuous community-based ITN distribution pilot in Lainya County, South Sudan 2012–2013. Malar J..

[26] Stuck L, Lutambi A, Chacky F, Schaettle P, Kramer K, Mandike R (2017). Can school-based distribution be used to maintain coverage of long-lasting insecticide-treated bed nets: evidence from a large scale programme in southern Tanzania?. Health Policy Plan..

[27] Smithuis FM, Kyaw MK, Phe UO, van der Broek I, Katterman N, Rogers C (2013). The effect of insecticide-treated bed nets on the incidence and prevalence of malaria in children in an area of unstable seasonal transmission in western Myanmar. Malar J..

[28] Pinchoff J, Hamapumbu H, Kobayashi T, Simubali L, Stevenson JC, Norris DE (2015). Factors associated with sustained use of long-lasting insecticide-treated nets following a reduction in malaria transmission in Southern Zambia. Am J Trop Med Hyg.

[29] Thwing J, Hochberg N, Eng JV, Issifi S, James Eliades M, Minkoulou E (2008). Insecticide-treated net ownership and usage in niger after a nationwide integrated campaign. Trop Med Int Health..

[30] Mabaso ML, Sharp B, Lengeler C (2004). Historical review of malarial control in southern African with emphasis on the use of indoor residual house-spraying. Trop Med Int Health..

